# Glutamatergic and GABAergic reactivity and cognition in 22q11.2 deletion syndrome and healthy volunteers: A randomized double-blind 7-Tesla pharmacological MRS study

**DOI:** 10.1177/0269881120922977

**Published:** 2020-05-25

**Authors:** Claudia Vingerhoets, Desmond HY Tse, Mathilde van Oudenaren, Dennis Hernaus, Esther van Duin, Janneke Zinkstok, Johannes G Ramaekers, Jacobus FA Jansen, Grainne McAlonan, Therese van Amelsvoort

**Affiliations:** 1 Department of Psychiatry & Neuropsychology, Maastricht University, Maastricht, the Netherlands; 2 Department of Radiology & Nuclear Medicine, Amsterdam University Medical Center, Location AMC, Amsterdam, the Netherlands; 3 Faculty of Psychology and Neuroscience, Maastricht University, Maastricht, the Netherlands; 4 Department of Psychiatry & UMC Utrecht Brain Center, University Medical Center, Utrecht, the Netherlands; 5 Department of Radiology, Maastricht University Medical Center, Maastricht University, Maastricht, the Netherlands; 6 The Sackler Institute for Translational Neurodevelopment, Institute of Psychiatry, Psychology and Neuroscience, King’s College London, London, UK

**Keywords:** Glutmate, GABA, 22q11.2DS, riluzole, cognition, ^1^H-MRS, voltage-gated sodium channel blocker

## Abstract

**Aims:**

22q11.2 deletion syndrome (22q11.2DS) is associated with impaired cognitive functioning. Glutamatergic pathways have been linked with cognition and are hypothesized to be disrupted in 22q11.2DS patients, possibly ‘shifting’ the excitatory (glutamate)/inhibitory (GABA) balance. Hence, the glutamate/GABA balance may constitute a target for pharmacological treatment. We aimed to examine alterations of glutamate/GABA metabolites in 22q11.2DS *in vivo* using riluzole, a compound with glutamate/GABA-modulating action, as pharmacological challenge.

**Methods:**

Seventeen 22q11.2DS patients and 20 matched healthy controls were enrolled in this randomized double-blind placebo-controlled crossover study. Glutamate and glutamine concentrations in the anterior cingulate cortex (ACC) and striatum, as well as ACC GABA concentrations were obtained after placebo and after a single dose of 50 mg riluzole using 7-Tesla magnetic resonance spectroscopy (MRS). Within the 22q11.2DS group, the relationship between metabolite concentrations and cognition was examined.

**Results:**

No group differences were found in ACC and striatal metabolite concentrations following placebo. Riluzole numerically decreased ACC (*η*^2^ *= 0.094*) but not striatal glutamate concentrations as well as ACC GABA concentrations (*η*^2^ *= 0.176*) in all subjects. In both regions, riluzole did not alter glutamine concentration. No interaction effects were found. Although not significant after Bonferroni correction, ACC glutamate concentrations were inversely correlated with cognitive functions in 22q11.2DS patients.

**Discussion:**

We did not demonstrate altered ACC and striatal metabolite concentrations in 22q11.2DS. Nevertheless, these results suggest that glutamate and GABA can be modulated with a single dose of riluzole. Possibly, riluzole may have memory-enhancing effects in 22q11.2DS. Future studies should examine the long-term effects of riluzole on cognition.

## Introduction

22q11.2 deletion syndrome (22q11.2DS), also referred to as velocardiofacial or DiGeorge syndrome, is a genetic disorder caused by a microdeletion on the long arm of chromosome 22 (Jonas et al., 2014) and is, with a prevalence of 1 in 2000–4000 births, one of the most common recurrent copy number variant disorders (Schneider et al., 2014). Its phenotypic expression is highly heterogeneous and includes medical conditions such as congenital heart disease, palatal anomalies, hypocalcaemia and dysmorphic facial features (Bassett et al., 2011). In addition, 22q11.2DS is associated with a high risk of developing psychiatric disorders, including psychosis spectrum disorders (Schneider et al., 2014) and the majority of 22q11.2DS patients have a below-average IQ and display impairments in cognitive functioning. Cognitive functioning often further declines with age and has been found to be steeper in 22q11.2DS patients developing psychosis (Vorstman et al., 2015).

The typically deleted region is 1.5–3 Mb (megabases) in size, including approximately 90 genes, of which most are expressed in the brain (Guna et al., 2015). One of the genes located in the deleted region is the proline dehydrogenase (*PRODH*) gene, which encodes the enzyme *PRODH* (also known as proline oxidase), which is important for breaking down proline. Proline is converted to glutamate and acts as a co-agonist at the glutamatergic n-methyl-d-aspartate (NMDA) receptor (Cohen and Nadler, 1997). Observations of increased proline levels in 22q11.2DS have been hypothesized to result from reduced *PRODH* enzyme activity in 22q11.2DS due to haploinsufficiency of the *PRODH* gene (Goodman et al., 2000; Vorstman et al., 2009). Decreased *PRODH* enzyme activity can thus lead to increased proline levels and, subsequently, increased activation of the NMDA receptor and excessive glutamate release (Cohen and Nadler, 1997; Evers et al., 2015; Magnée et al., 2011; Paterlini et al., 2005). Glutamate is the primary excitatory neurotransmitter and is hypothesized to be involved in the pathophysiology of psychosis, with the exception of the rare NMDA-receptor encephalitis disorder and drug states, as well as in cognitive functioning (Lewis and Moghaddam, 2006). Excessive glutamate concentrations are toxic for the brain as this can result in cell death (Lau and Tymianski, 2010). Thus, it could be hypothesized that cognitive decline often observed in 22q11.2DS (Vorstman et al., 2015) is related to prolonged, increased glutamate levels.

Glutamate function is closely correlated with y-aminobutyric acid (GABA): the main inhibitory neurotransmitter in the brain. For example, activity of cortical GABA neurons is partly regulated by glutamatergic inputs (Lewis and Moghaddam, 2006; Marsman et al., 2014). Yet, both systems are often studied in isolation and little is known about GABA in 22q11.2DS. Neuroprotective drugs that modulate glutamatergic neurotransmission and restore the glutamate/GABA balance may effectively enhance cognitive functioning in patients with 22q11.2DS, and possibly reduce disease-associated cognitive decline. One potential candidate drug is riluzole, a Food and Drug Administration-approved glutamate and GABA-modulating compound. Riluzole has neuroprotective properties (Doble, 1996) and is clinically used for the treatment of amytrophic lateral sclerosis (ALS). Riluzole is a potent antiglutamatergic agent that reduces glutamatergic neurotransmission via several mechanisms of action, including inhibition of presynaptic glutamate release through inactivation of voltage-dependent sodium channels on glutamatergic nerve terminals and calcium currents (Bellingham, 2011; Doble, 1996). Furthermore, riluzole enhances astrocytic glutamate reuptake and reduces the amount of releasable presynaptic glutamate (Lazarevic et al., 2018). In addition, riluzole stimulates GABAergic neurotransmission by stimulating GABA_A_ receptor potentiation and blocking its reuptake (He et al., 2002; Jahn et al., 2008). In other words, riluzole has the potential to restore the glutamate/GABA-balance. Importantly, riluzole has a well-established pharmacokinetic and safety profile, has a low risk of adverse effects, and has been found to be well tolerated in several psychiatric diseases (de Boer et al., 2019; Mathew et al., 2017; Pittenger et al., 2015; Zarate, 2008). Therefore, the aim of the present study was to (a) compare brain concentrations of glutamatergic metabolites (glutamate and glutamine) between patients with 22q11.2DS and controls, (b) to examine the effects of riluzole on these metabolites and (c) within 22q11.2DS to examine the relationship between these metabolites and cognitive functioning. In addition, we explored whether GABA concentrations in the ACC differ between patients and controls, and whether riluzole modulated GABA levels in both groups.

## Methods

This study was approved by the Medical Ethical Committee of Maastricht University in the Netherlands (METC142046, NL49834.068.14). All participants gave written informed consent following a full explanation of the study procedure. This study was registered in the Netherlands Trial Register (NTR5095).

### Participants

Seventeen 22q11.2DS patients without a history of psychosis, and 20 age and gender matched healthy controls were enrolled in the study. All participants were free of antipsychotic medication at time of scanning. Patients with 22q11.2DS were recruited through the 22q11 outpatient clinic of the academic hospital Maastricht (azM) and through family associations. Healthy participants were recruited via advertisement on the internet. Inclusion criteria were, aged between 18 and 65 years, and for adults with 22q11.2DS, a confirmed diagnosis of 22q11.2DS established by fluorescence *in situ* hybridization, microarray or multiplex ligation-dependent probe amplification, and the mental capacity to give informed consent. Exclusion criteria for both groups were a history of severe psychiatric or neurological disorders, contraindications for magnetic resonance imaging (MRI) or riluzole and recreational drug use 4 weeks prior to participation. For female participants, pregnancy was an additional exclusion criterion and was verified with a urine screening. All participants were instructed to refrain from alcohol and nicotine 24 h before testing.

### Instruments

The full scale intelligence quotient was estimated with a shortened version of the Wechsler Adult Intelligence Scale, version 3 (WAIS-III, (Velthorst et al., 2012)). The Mini International Neuropsychiatric Interview (Sheehan et al., 1997) was used to verify absence of psychiatric disorders. Potential side effects of riluzole were measured with a standardized 31-item self-report inventory using a 4-point Likert scale (0 not affected–3 very affected) (Wezenberg et al., 2005). Cognitive performance was measured with the Cambridge Neuropsychological Test Automated Battery (CANTAB, schizophrenia test battery) (Levaux et al., 2007). This test battery has been well validated and is regarded the ‘gold standard’ of cognitive assessment. CANTAB has been found to be sensitive for pharmacological agents (Barnett et al., 2010) and the sensitivity of the subtests enables detection of subtle effects. We administered the following subtests: Paired Associate Learning, Verbal Recognition Memory, Spatial Working Memory, and Rapid Visual Processing, which cover visual learning and memory, verbal learning and memory, attention and vigilance and working memory, respectively.

### ^1^H magnetic resonance spectroscopy

**Figure 1.**
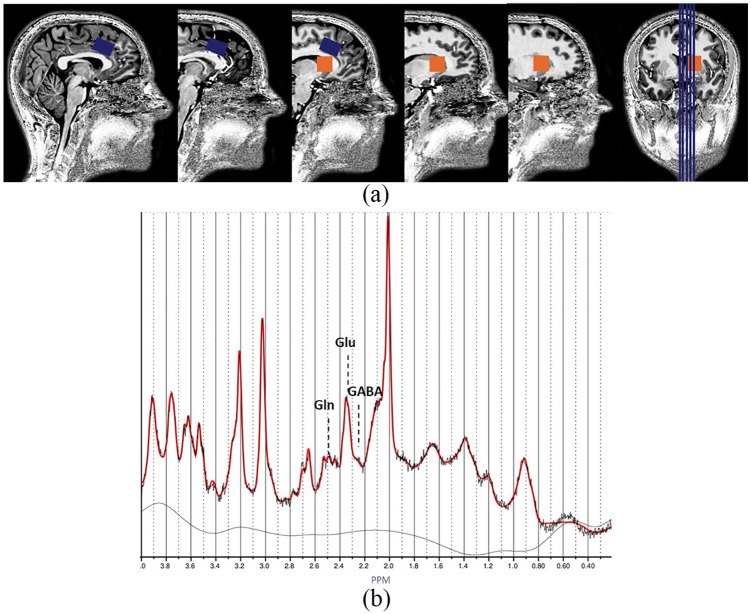
(a) Voxel placement. The blue box indicates the location of the ACC voxel box and orange indicates the location of the striatal voxel. (b) Example of an ACC spectrum derived from a healthy control. PPM: parts per million.

Single-voxel proton magnetic resonance spectroscopy (1H-MRS) measurements were performed on a MAGNETOM 7T MR scanner (Siemens Healthineers, Erlangen, Germany) using a single-channel transmit/32-channel receiving head coil (Nova Medical, Wilmington, MA, USA). Spectra were acquired with a stimulated echo acquisition mode (STEAM) (Frahm et al., 1987) sequence using the following parameters: TE = 6.0 ms, TM = 10.0 ms, TR = 5.0 s, NA = 64, flip angle = 90°, radio frequency (RF) bandwidth = 4.69 kHz, RF centred at 2.4 ppm, receive bandwidth = 4.0 kHz, vector size = 2048, 16-step phase cycling, acquisition time = 5:20 min. Water suppression was achieved by variable power RF pulses with optimized relaxation delays (Tkáč et al., 1999). In addition, a complete phase cycle of measurements was acquired without the water suppression RF pulses, to record a water peak reference for eddy current correction (Klose, 1990) and absolute metabolite concentration calibration (Barker et al., 1993; Soher et al., 1996). Spectroscopic voxels of interest were manually placed by a trained operator at the anterior cingulate cortex (ACC) (voxel size = 25 × 20 × 17 mm^3^) and the right striatum (voxel size = 20 × 20 × 20 mm^3^) (Figure 1). Prior to the spectroscopy measurements, a 3D-GRE dual-echo field map (TE_1_ = 1.00 ms, TE_2_ = 2.98 ms, TR = 20.0 ms, flip angle = 8°, voxel size = 3 mm isotropic, matrix size = 84 × 84 × 56, bandwidth = 1450 Hz/pixel, acquisition time = 2:24 min) was acquired and used to calculate the shim currents required to homogenise the static magnetic field in the spectroscopic voxels of interest. In addition, an anatomical (T_1_-weighted) image was acquired using magnetization-prepared two rapid acquisition gradient-echo (MP2RAGE) (Marques et al., 2010) sequence (TR = 4.5 s, TE = 2.39 ms, TI_1_ = 0.90 s, TI_2_ = 2.75 s, flip angle_1_ = 5°, flip angle_2_ = 3°, voxel size = 0.9 mm isotropic, matrix size = 256 × 256 × 192, phase partial Fourier = 6/8, GRAPPA factor = 3 with 24 reference lines, bandwidth = 250 Hz/pixel, acquisition time = 6:00 min). The spectra were analysed with LCModel version 6.3-1L (Provencher, 2001) using a GAMMA-simulated basis set (Smith et al., 1994). Metabolite concentrations were excluded from statistical analysis when the Cramer–Rao lower bound exceeded 20%. Spectral quality per group (signal to noise ratio and full width at half maximum) is displayed in Supplementary Table 2. Tissue probability maps for grey matter (GM), white matter (WM) and cerebrospinal fluid (CSF) were generated from the T_1_-weighted anatomical images using FSL-FAST (Zhang et al., 2015). GM, WM and CSF partial volumes within the spectroscopy voxels were estimated from these tissue probability maps. Metabolite concentrations were corrected for proportion of CSF as described in Quadrelli et al. (2016).

### Procedure

This study had a randomized double-blind placebo-controlled crossover design. Participants were randomly allocated to either receive placebo or riluzole on the first visit. Ten 22q11.2DS patients and 11 controls received placebo during the first visit. Blinding was done by a research associate who was not involved in any study procedures and both researchers and participants were blind to the order of the intervention. All participants underwent ^1^H-MRS measurements twice: once following placebo and once following oral administration of 50 mg riluzole. This dose was chosen as we were interested in the acute effects and to minimize the burden for participants. To assure all participants were free of recreational drugs, a urine drug screen for cannabis, cocaine, benzodiazepines, opiates, amphetamines and methamphetamines was conducted at the start of each test day. None of the participants tested positive on any of the testing days. Furthermore, no female participants tested positive for pregnancy in a second urine screening. Both testing days started with a brief explanation of the procedure after which the study medication was administered under supervision of the test leader. Scanning commenced 90 min after drug administration given that riluzole reaches T_max_ approximately 60–90 min after oral administration (Zarate, 2008). To monitor potential adverse effects, a self-report inventory was completed 5 min before scanning. The two scan sessions were separated by at least 1 week to ensure complete drug wash out. The CANTAB and the WAIS-III were always conducted on the first day prior to drug administration, to prevent possible confounding effects of riluzole.

#### Statistical analyses

All statistical analyses were performed with IBM SPSS Statistics, version 25. First, differences in sample demographics including sex, age, IQ and adverse effects were examined using chi-square or Mann–Whitney U tests respectively. Second, voxel composition was compared between groups using independent sample *t*-tests (Supplementary Table 1). Group differences in ACC and striatum metabolite concentrations collected during the placebo session were compared (glutamate, glutamine, GABA) using independent sample *t*-tests. Drug-induced change in metabolite concentrations, as well as group-by-drug interaction, was examined using a repeated measures analyses of variance (rmANOVA). Finally, we examined whether metabolite concentrations were associated with separate cognitive domains (visual memory, verbal memory, working memory and attention) using Spearman’s correlation coefficient as this measure is more robust to the influence of outliers compared to other correlation coefficients (King, 1992; Pillinger et al., 2019). For this purpose, cognitive domain scores were computed for the four cognitive domains (visual memory, verbal memory, working memory, attention). First, the raw scores were converted to standardized *Z*-scores. The domains for which a lower score represents better performance, were reversed-scored so that a higher score represented better performance for all domains. Bonferroni correction was applied to correct for multiple comparisons (0.05/(3 (metabolites) × 2 (brain regions) × 4 (cognitive domains)). Consequently, a *p*-value ⩽ 0.002 was considered significant.

## Results

### Demographics

Sample demographics are displayed in Table 1. No between-group differences were observed for age (*t*(1,36) = −1.05, *p* = 0.304) and sex (*χ*^2^(1) = 0.09, *p* = 0.769). Patients with 22q11.2DS had a significantly lower IQ compared with healthy controls (*t*(1,36) = 8.99, *p* < 0.001). Riluzole and placebo session side effect scores did not differ across the entire sample (*U* = 113.0, *p* = 0.186).

**Table 1.** Sample demographics.

	HC	22q11.2DS	Statistic	*p*-value
	Mean (SD)	Mean (SD)		
Sex (m/f)	8/12	6/11	0.09	0.769
Age	30.7 (8.20)	34.17 (11.41)	−1.05	0.304
FSIQ	120.2 (16.23)	76.65 (12.32)	8.99	**<0.001**
Side effects				
Placebo	3.42 (4.7)	3.50 (3.1)	128.5	0.428
Riluzole	2.42 (4.1)	4.25 (4.3)	113.0	0.186
SSRI use (no/yes)	19/1	15/2	0.56	0.452

Note: FSIQ: full scale intelligence quotient; SSRI: selective serotonin reuptake inhibitor; HC: healthy controls. Significant results are bold.

### ^1^H-MRS metabolite concentrations

**Figure 2.**
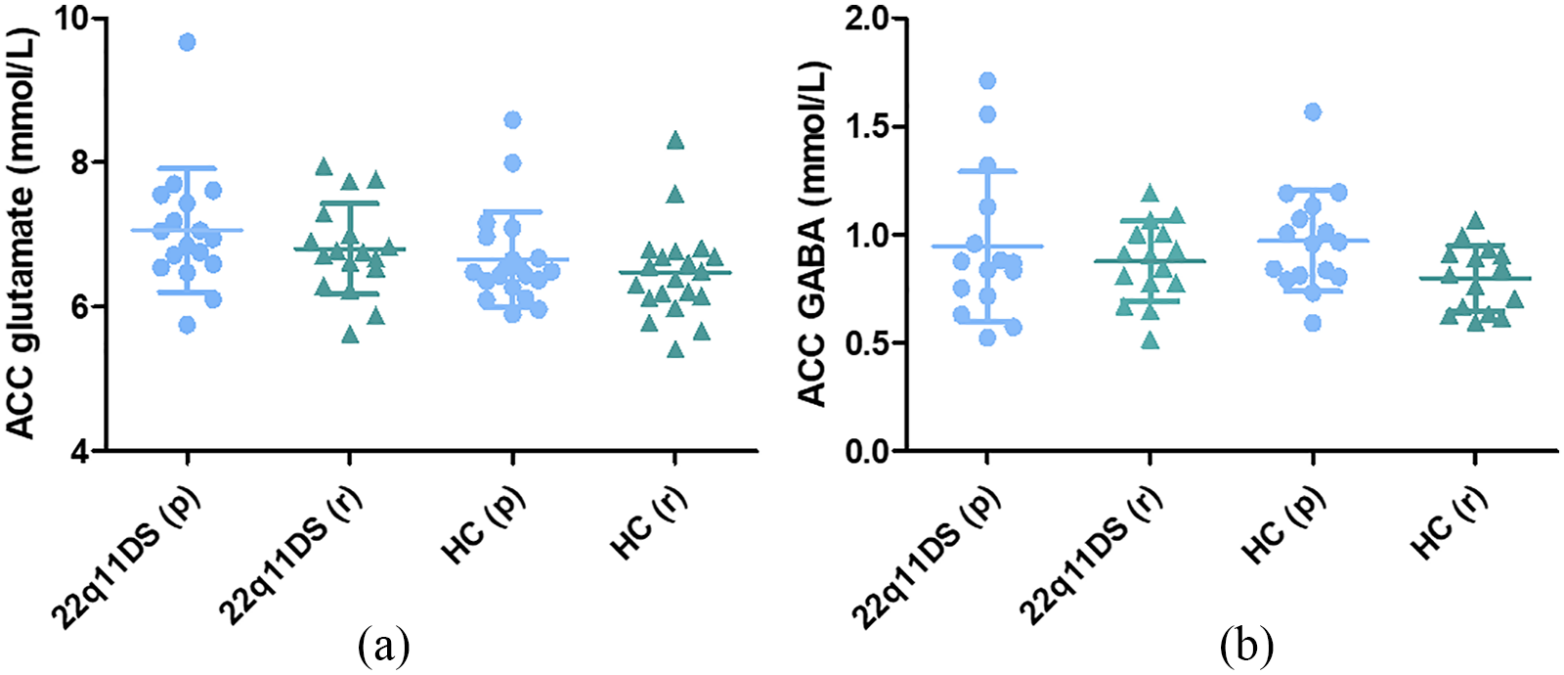
**(a)** Glutamate concentrations in the anterior cingulate cortex (ACC). Riluzole decreased ACC glutamate concentrations at trend level (*F*(1,35) = 3.61, *p* = 0.066, *η*^2^ = 0.094) in both groups. (b**)** GABA concentrations in the ACC. Riluzole decreased ACC GABA levels in both groups (*F*(1,24) = 5.14, *p* = 0.033, *η*^2^ = 0.176). Whiskers in (a) and (b) indicate minimum and maximum values.

We did not find differences in metabolite concentrations after placebo between groups (Table 2). However, glutamate concentrations were numerically higher in 22q11.2DS patients with an effect size in the medium range (*d* = 0.528). We observed a trend in decreased ACC glutamate concentrations after riluzole administration in the total sample (Figure 2a), although this effect did not reach significance despite a medium–high effect size (*F*(1,35) = 3.61, *p* = 0.066, *η*^2^ = 0.094). No group-by-drug interaction effect was observed (*F*(1,35) = 0.11, *p* = 0.738, *η*^2^ = 0.003). No evidence for a main effect of riluzole on striatal glutamate concentrations was observed (*F*(1,34) = 0.19, *p* = 0.669, *η*^2^ = 0.005), nor was a group-by-drug interaction (*F*(1,34) = 0.02, *p* = 0.904, *η*^2^ = 0.000). No main or group-by-drug interaction effect of riluzole was observed for either ACC or striatal glutamine concentrations (Table 3). Finally, GABA concentrations in the ACC decreased in both groups after riluzole administration (*F*(1,24) = 5.14, *p* = 0.033, *η*^2^ = 0.176, Figure 2b). No group-by-drug interaction was found for ACC GABA levels (*F*(1,24) = 0.28, *p* = 0.603, *η*^2^ = 0.011).

**Table 2.** Baseline metabolite concentrations, CSF corrected.

		HC		22q11.2DS	CRLB in %	95% CI		
		Mean (SD)		Mean (SD)	Mean (SD)	lower–upper	*p*	*d*
ACC	*N*		*N*					
Glutamate	20	6.659 (0.66)	17	7.061 (0.85)	2.3 (0.45)	−0.90 to 0.10	0.115	0.528
GABA	15	0.930 (0.18)	14	0.890 (0.28)	11.7 (3.42)	−0.14 to 0.22	0.651	0.084
Glutamine	12	0.816 (0.20)	14	0.801 (0.23)	9.1 (2.69)	−0.13 to 0.16	0.834	0.070
Striatum								
Glutamate	20	4.419 (0.63)	16	4.628 (0.80)	4.2 (1.62)	−0.68 to 0.27	0.380	0.290
Glutamine	13	1.181 (0.32)	12	1.132 (0.42)	11.1 (2.92)	−0.23 to 0.32	0.717	0.131

Note: ACC: anterior cingulate cortex; CSF: cerebral spinal fluid; CI: confidence interval for difference; CRLB: Cramer–Rao lower bound.

**Table 3.** Effects of riluzole on brain metabolites.

		Healthy controls			22q11.2DS						
		Placebo	Riluzole		Placebo	Riluzole	95% CI	Main effect riluzole		Group × medication interaction	
	*N*	Mean (SD)	Mean (SD)	*N*	Mean (SD)	Mean (SD)	Lower–upper	*P*	*η* ^2^	*P*	*η* ^2^
ACC											
Glutamate	20	6.659 (0.66)	6.477 (0.64)	17	7.061 (0.85)	6.800 (0.63)	−0.015 to 0.458	0.066	0.094	0.738	0.003
Glutamine	19	0.816 (0.20)	0.832 (0.23)	16	0.785 (0.22)	0.794 (0.25)	−0.085 to 0.059	0.712	0.004	0.928	0.000
GABA	12	0.938 (0.25)	0.775 (0.16)	14	0.975 (0.34)	0.874 (0.19)	−0.023 to 0.195	**0.033**	0.176	0.603	0.011
Striatum											
Glutamate	20	4.419 (0.63)	4.389 (0.70)	16	4.674 (0.80)	4.620 (0.76)	−0.691 to 0.205	0.669	0.005	0.904	0.000
Glutamine	13	1.151 (0.32)	1.137 (0.36)	12	1.025 (0.23)	0.950 (0.30)	−0.024 to 0.113	0.196	0.072	0.370	0.035

Note: ACC: anterior cingulate cortex; CI: confidence interval for difference. Significant effects are bold.

### Relationship with cognition

Within the 22q11.2DS group, glutamate levels in the ACC after placebo were inversely correlated with visual memory (*r* = −0.593, *p* = 0.012) and verbal memory (*r* = −0.510, *p* = 0.036). ACC GABA levels under placebo were inversely correlated with attention (*r* = −0.525, *p* = 0.044). However, after Bonferroni correction, none of the associations remained significant.

## Discussion

Here, we examined whether ACC and striatal glutamatergic metabolite concentrations are altered in adults with 22q11.2DS and if a single dose of riluzole modulates these metabolites. Despite a medium effect size, we found no group differences in ACC and striatal metabolite concentrations following placebo administration. This is in line with studies by Rogdaki et al. (2018) and da Silva Alves et al. (2011), neither of whom found differences in frontal levels of the glutamate + glutamine complex (Glx) between patients with 22q11.2DS and healthy controls. Yet, da Silva Alves et al. (2011) did report higher hippocampal Glx concentrations in 22q11.2DS patients with a psychotic disorder compared with 22q11.2DS patients without psychosis and healthy controls, consistent with findings of higher frontal Glx levels in patients with psychosis without 22q11.2DS (Merritt et al., 2016). This may indicate that glutamatergic alterations may be a ‘state’ rather than a ‘trait’ characteristic of psychosis (Rogdaki et al., 2018) and/or may be region specific. However, both Rogdaki et al. (2018) and da Silva Alves et al. (2011) used 3-Tesla MRI and therefore were not able to differentiate between glutamate and glutamine. Using 7-Tesla we were able to measure both glutamate and glutamine separately due to increased spatial and spectral resolution at 7-Tesla (Mekle et al., 2009). Given that our sample was small, and the *p*-value relatively low with a corresponding medium effect size, we cannot exclude the possibility that glutamate levels (rather than glutamine) may be marginally heightened in 22q11.2DS without psychosis.

Interestingly, we observed a trend in decreased glutamate levels after riluzole in the ACC, but not striatal, glutamate concentrations, whereas no effect of riluzole on glutamine was found in either brain regions. Although not reaching significance in this small sample, the medium to large effect size implies that riluzole is able to modulate glutamate neurotransmission, even after a single administration. Moreover, our results suggest that it primarily targets glutamate rather than other glutamatergic metabolites. Furthermore, we found that higher ACC glutamate concentrations in the placebo condition were associated with poorer visual and verbal memory performance in 22q11.2DS (although not significant after Bonferroni correction), implicating that glutamatergic neurotransmission may be involved in some of the cognitive deficits observed in 22q11.2DS. Although not significant after Bonferroni correction, GABA levels were also inversely associated with memory and attention. In line with findings in other neurological/psychiatric disorders including psychosis (Lewis and Moghaddam, 2006; Merritt et al., 2013; Taylor and Tso, 2015; Vingerhoets et al., 2013), this confirms a role of glutamate and GABA in cognitive impairments in 22q11.2DS. Modulation of glutamate/GABA neurotransmission may therefore be a target for pharmacological treatment of cognitive symptoms in patients with 22q11.2DS. Since our results suggest that riluzole modulates both glutamate and GABA in 22q11.2DS, this compound may be an effective treatment for cognitive impairment in 22q11.2DS. Indeed, improvement in memory and attention was found in a female patient with 22q11.2DS after 18 months of treatment with riluzole (Vingerhoets et al., 2019). Unfortunately, in this study we only used a single dose of riluzole and we only conducted cognitive tests at baseline in order to minimize the burden for participants. Hence, future studies examining cognition-enhancing effects of long-term riluzole treatment are warranted.

A study by Pillinger et al. (2019) reported decreased ACC Glx concentrations in treatment-resistant schizophrenia patients following a 2-day challenge with 50 mg riluzole twice daily using 3T MRS, whereas we found a trend for reduced glutamate but not glutamine concentrations following a single dose of riluzole. In addition, Brennan et al. (2010) reported an increased glutamine/glutamate ratio in bipolar depression after a similar 2-day riluzole challenge using 3T MRS. However, both these studies used a repeated administration design whereas we only administered a single (low) dose of riluzole. A possible explanation for the divergent results is that changes in glutamate and/or glutamine only occur after multiple and/or higher riluzole doses. Another possibility is that riluzole has different effects in pathological and non-pathological conditions, as both Pillinger et al. (2019) and Brennan et al. (2010) included individuals with a psychiatric disorder, whereas our 22q11.2DS sample had no history of psychiatric disorders. Moreover, in contrast to our sample, patients in both studies were using antipsychotic or antidepressant medication, which could have interacted with riluzole’s mechanisms of action. It is worth mentioning that both previous studies were conducted at lower MR field strength and consequently were not able to reliably distinguish glutamate and glutamine. Previous results obtained at 3T may therefore reflect changes in glutamate rather than glutamine. Indeed, when looking at the effect sizes, we observed a medium to large effect of riluzole on ACC glutamate concentrations, whereas its effect on glutamine in this region appears to be small. Contrarily, we found a medium effect size of riluzole on striatal glutamine whereas the effect on striatal glutamate was small. This could indicate that riluzole’s mechanism of action differs between cortical and subcortical regions. This would seem consistent with the observation that, in rodents, a decrease in striatal glutamate was only observed at high riluzole doses, whereas a decrease in prefrontal cortex glutamate concentrations was already visible after a low dose of riluzole (Waschkies et al., 2014). In line with our results, no changes were observed in glutamine concentrations in either region at low, medium or high dosages (Waschkies et al., 2014).

Finally, exploratory analyses showed a decrease (main effect of drug) in ACC GABA concentrations after riluzole administration. The mechanism of action of riluzole is complex and currently, not completely understood. Nonetheless, although riluzole preferentially inhibits glutamate release, it has also been reported to influence GABAergic transmission (Jahn et al., 2008; Jehle et al., 2000). While both pre- and postsynaptic effects of riluzole on GABAergic transmission have been established, postsynaptic potentiating effects of riluzole on GABA_A_ receptors have only been reported at higher dosages (He et al., 2002). This could be a possible explanation for the decrease in ACC GABA concentrations given that the dosage used in this study was relatively low. Indeed Ajram et al. (2017) reported decreased prefrontal GABA concentrations in healthy controls following the same dose of riluzole, and an increase in patients with autism spectrum disorders. Unfortunately, to the best of our knowledge, no other *in vivo* studies examining effects of a higher dosage of riluzole on GABA concentrations in humans have been conducted.

### Strengths and limitations

A major strength of this study is the use of 7T MRI. At 3T, glutamate and glutamine cannot be reliably distinguished and are therefore typically measured as the Glx. Due to increased spectral resolution at 7T we were able to reliably obtain separate measures of glutamate and glutamine. Moreover, 7T allows for measurements of GABA using a STEAM sequence (Wijtenburg et al., 2013). Nonetheless, our protocol was not optimized for quantification of GABA resulting in relatively higher variance of GABA concentrations (Table 2). Therefore, results regarding GABA should be considered exploratory and interpreted with care. Future studies could use a separate sequence optimized for measuring GABA to further reduce variance.

Furthermore, subjects had no diagnosis of a psychotic disorder and were antipsychotic naïve at time of inclusion. Since psychotic disorders are also associated with glutamatergic alterations, our results reflect glutamatergic alterations related to the 22q11.2 deletion. Another potential drawback may be that inclusion criteria for 7T MRI are stringent and no implants are allowed. Many patients with 22q11.2DS carry metal implants due to congenital heart problems or scoliosis. This may have caused a selection bias of relatively healthy patients. Furthermore, the sample size of our study was relatively small, resulting in limited power to detect group differences and may explain the lack of statistically significant group differences. However, the medium to large effect sizes suggest that results may have reached significance in a bigger sample. Another limitation is that we used a single, relatively low dose of riluzole, which could explain the lack of a significant effect of riluzole on glutamate. The recommended daily dosage in ALS is 100 mg (50 mg twice daily) (Bruno et al., 1997). We chose this dose as we were interested in acute effects and wanted to minimize the burden for the participants. However, other studies in treatment-resistant schizophrenia (Pillinger et al., 2019) and depression (Brennan et al., 2010) showed a decrease in glutamate concentrations after a 2-day challenge with 100 mg riluzole. Future studies should examine the effects of long-term riluzole treatment in a larger sample of 22q11.2DS patients. With regard to ^1^H-MRS, an important limitation of this method is that it does not enable precise localization of glutamatergic metabolites (e.g. intracellular vs. extracellular and pre- vs. postsynaptic).

In conclusion, we demonstrated that a single dose of riluzole decreases ACC glutamate concentrations at trend level in 22q11.2DS. Given the inverse correlation between glutamate and memory in 22q11.2DS, modulation of the glutamate/GABA system using riluzole may have cognitive-enhancing effects in 22q11.2DS. Given glutamate’s role in psychotic disorders and findings of increased Glx levels in psychosis, future studies should examine whether riluzole treatment could also enhance cognition in a bigger sample of 22q11.2DS patients. However, more studies are required to replicate these findings and to examine the effects of long-term treatment with riluzole.

## Supplemental Material

Supplementary_material_riluzole_final_JoP_revised – Supplemental material for Glutamatergic and GABAergic reactivity and cognition in 22q11.2 deletion syndrome and healthy volunteers: A randomized double-blind 7-Tesla pharmacological MRS study Click here for additional data file. Supplemental material, Supplementary_material_riluzole_final_JoP_revised for Glutamatergic and GABAergic reactivity and cognition in 22q11.2 deletion syndrome and healthy volunteers: A randomized double-blind 7-Tesla pharmacological MRS study by Claudia Vingerhoets, Desmond HY Tse, Mathilde van Oudenaren, Dennis Hernaus, Esther van Duin, Janneke Zinkstok, Johannes G Ramaekers, Jacobus FA Jansen, Grainne McAlonan and Therese van Amelsvoort in Journal of Psychopharmacology
