# Supplementary material for: Glutamatergic and GABAergic reactivity and cognition in 22q11.2 deletion syndrome and healthy volunteers: A randomized double-blind 7-Tesla pharmacological MRS study
Source: J Psychopharmacol. 2020 May 25;34(8):856–63. doi: 10.1177/0269881120922977 (PMC7376622; doi:10.1177/0269881120922977)
Supplement: Supplementary_material_riluzole_final_JoP_revised – Supplemental material for Glutamatergic and GABAergic reactivity and cognition in 22q11.2 deletion syndrome and healthy volunteers: A randomized double-blind 7-Tesla pharmacological MRS study [file Supplementary_material_riluzole_final_JoP_revised.pdf]

Supplementary material

Supplementary table 1. voxel composition.

	Healthy controls	22q11.2DS		
	Mean (SD)	Mean (SD)	*t*-value	*p*
**Placebo**				
ACC GM	0.532 (0.037)	0.528 (0.064)	0.193	0.848
ACC WM	0.241 (0.029)	0.240 (0.063)	0.020	0.984
ACC CSF	0.228 (0.045)	0.231 (0.073)	-0.152	0.880
Striatum GM	0.432 (0.044)	0.467 (0.056)	-1.501	0.142
Striatum WM	0.481 (0.045)	0.458 (0.053)	1.409	0.168
Striatum CSF	0.087 (0.041)	0.085 (0.052)	0.125	0.901
**Riluzole**				
ACC GM	0.538 (0.034)	0.513 (0.060)	1.494	0.128
ACC WM	0.242 (0.030)	0.250 (0.052)	-0.540	0.594
ACC CSF	0.220 (0.037)	0.237 (0.075)	-0.834	0.413
Striatum GM	0.437 (0.040)	0.436 (0.051)	0.068	0.946
Striatum WM	0.484 (0.049)	0.463 (0.064)	1.084	0.286
Striatum CSF	0.079 (0.036)	0.100 (0.044)	-1.619	0.115

NB: ACC: anterior cingulate cortex; GM: grey matter; WM: white matter; CSF: cerebral spinal fluid.

Supplementary table 2. Spectral quality per group.

	ACC				Striatum			
	Placebo Mean (SD)		Riluzole Mean (SD)		Placebo Mean (SD)		Riluzole Mean (SD)	
	22q11.2DS	HC	22q11.2DS	HC	22q11.2DS	HC	22q11.2DS	HC
FWHM	0.031 (0.008)	0.035 (0.007)	0.034 (0.008)	0.037 (0.011)	0.080 (0.031))	0.073 (0.016)	0.065 (0.014)	0.076 (0.020)
SNR	40 (5.5)	36 (6.7)	38 (6.8)	37 (5.3)	23 (4.8)	22 (5.1)	24 (5.6)	21 (5.7)

NB: FWHM: full width at half maximum (linewidth); SNR: Signal Noise Ratio.
